# Effectiveness of gel formulation of capa leaf (*Blumea balsamifera* L.) on wound healing in white rats

**DOI:** 10.14202/vetworld.2022.2059-2066

**Published:** 2022-08-25

**Authors:** Masyudi Masyudi, Muhammad Hanafiah, Said Usman, Marlina Marlina

**Affiliations:** 1 Department of Medical Sciences, Graduate School of Mathematics and Applied Sciences, Universitas Syiah Kuala, Banda Aceh, Indonesia; 2 Department of Public Health, Universitas Serambi Mekkah, Banda Aceh, Indonesia; 3 Parasitology Laboratory, Faculty of Veterinary Medicine, Universitas Syiah Kuala, Banda Aceh, Indonesia; 4 Deapartment of Public Health, Faculty of Medicine, Universitas Syiah Kuala, Banda Aceh, Indonesia; 5 Department of Medical and Surgical Nursing, Faculty of Nursing, Universitas Syiah Kuala, Banda Aceh, Indonesia

**Keywords:** fibroblast, interleukin 2, propylene glycol, wound healing

## Abstract

**Background and Aim:**

The capa plant (*Blumea balsamifera* L.) has been widely used as a traditional herbal medicine in many parts of the world, including South Aceh, Indonesia. It is generally used for wound healing due to its antibacterial and anti-inflammatory properties. However, it is only available as extract or oil, and no gel formulation exists so far. Thus, in this study, we formulated the extract into a pharmaceutical gel and investigated its effectiveness in healing incision wounds in white rats (*Rattus norvegicus*).

**Materials and Methods:**

We collected *B. balsamifera* leaf samples from Gunongpulo village, South Aceh, Indonesia. We then produced leaf extract through maceration and formulated the extract into a gel using Carbopol 940, methylparaben, triethanolamine, and propylene glycol. We applied the gel to incision wounds in white rats for 7 and 14 days. We then monitored wound healing based on wound length, histology of skin tissues, and levels of cytokine 2 (interleukin-2 [IL-2]).

**Results:**

The gel formulation K3 (10% *B. balsamifera* leaf extract) was the most effective, followed by the gel formulations K2 (5% *B. balsamifera* leaf extract) and K4 (1% gentamicin ointment, positive control). K3 reduced wound length by 14 mm on day 7 and 29 mm on day 14. Histological analysis showed that fibroblast growth and angiogenesis were most significant in the K3-treated group, exceeding that of the positive control group. The K3-treated group also had the highest IL-2 levels, with an average of 107.7767 ng/L on day 7 and 119.1900 ng/L on day 14.

**Conclusion:**

The 10% *B. balsamifera* leaf gel effectively reduced wound length, increased fibroblast cell growth and angiogenesis, and IL-2 levels, accelerating wound healing.

## Introduction

Many parts of the world, including Indonesia, recognize herbal compounds as key traditional medicine ingredients [1–3]. The World Health Organization reported that numerous people use traditional herbal medicines for their primary healthcare [4]. The medicinal properties of plants are attributable to physiologically active biochemical compounds called secondary metabolites [4–6]. The pharmaceutical industry has greatly invested in chemistry and pharmacology research in the past two decades to find more effective medicines [7, 8]. Herbal compounds remain primary therapeutic agents in traditional and modern medicine [9]. The main reasons for sustainable use of herbal medicines are their usefulness, easy availability, low price, and relatively low or nonexistent toxicity [10]. They can be as potent as pharmaceutical drugs and help the body initiate self-healing [11].

The previous studies isolated bioactive compounds from the *Blumea balsamifera* leaf and documented their pharmacological properties. In addition, phytochemical components have been utilized as therapeutic agents [12, 13]. Besides, more complex semisynthetic chemical compounds are also derived from phytochemical compounds. Overall, the demand for medicinal plants increases, encouraging the exploration of new sources [14, 15].

*B. balsamifera* is a species used in traditional medicine in Indonesia, China, the Philippines, Thailand, Malaysia, and Vietnam. It is the most valuable member of the genus *Blumea* and is native to tropical and subtropical countries. Its small trees can grow up to 3–4 m on roadsides, fields, forest edges, under forests, lowlands, riverbeds, grasses, valleys, and mountainous areas. It also emits a strong camphor aroma [16, 17]. It contains flavonoids, sesquiterpenes, and triterpenoids [17–19]. The *B. balsamifera* plant oil (2000 mg/kg body weight) applied topically to the skin of white rats did not cause any allergic reactions or toxicity. In addition, *B. balsamifera* extracts can accelerate blood clotting [20]. They are also anti-inflammatory. The *B. balsamifera* extract using water as a solvent at a dose of 92 mg/kg body weight can reduce edema by up to 6.4% [21]. Moreover, *B. balsamifera* leaves from Aceh contain borneol, jasmoline, caryopeline, and several other flavonoids, which play a role in wound healing [22].

Gels are a common pharmaceutical formulation for topical drugs [23]. Topical gels present several advantages. First, their texture can increase comfort for the users. They can also be applied easily and evenly to the skin. Furthermore, they provide a cooling sensation, are well absorbed, and do not scar the skin [24]. Gels or jellies are systems with a semi-solid form made of an inorganic or organic suspension penetrated by a liquid. They offer more advantages than semi-solid forms, such as easy application and durability [25]. Besides, moisturizing a wound improves tissue growth, accelerating the wound healing process [26–28]. Therefore, formulating the active compounds in *B. balsamifera* leaves as a gel could make it more practical to apply and heal wounds faster.

*B. balsamifera* has been used as a raw material in traditional medicine [16]. Moreover, in the Aceh Province (especially South Aceh), *B. balsamifera* has been traditionally used for its wound healing [3], anti-inflammatory, and antibacterial properties [20]. No study has ever formulated *B. balsamifera* into a pharmaceutical gel, tested it on incision wounds, and evaluated its effectiveness in wound healing using three parameters simultaneously: Macroscopic analysis, histological analysis, and interleukin-2 (IL-2) expression.

In this study, we formulated the active compounds of the *B. balsamifera* leaf into a gel and applied it to incision wounds of white rats divided into five treatment groups. Next, we monitored wound healing to determine the best concentration of *B. balsamifera* leaf extract for wound care. To that end, we measured the average reduction in wound length, assessed fibroblast cell growth and angiogenesis, and quantified IL-2.

## Materials and Methods

### Ethical approval

This study was approved by the Veterinary Ethics Committee Faculty of Veterinary Medicine, Universitas Syiah Kuala, Banda Aceh-Indonesia (Approval No. 79/KEPH/XII/2020).

### Study period and location

**Figure-1:**
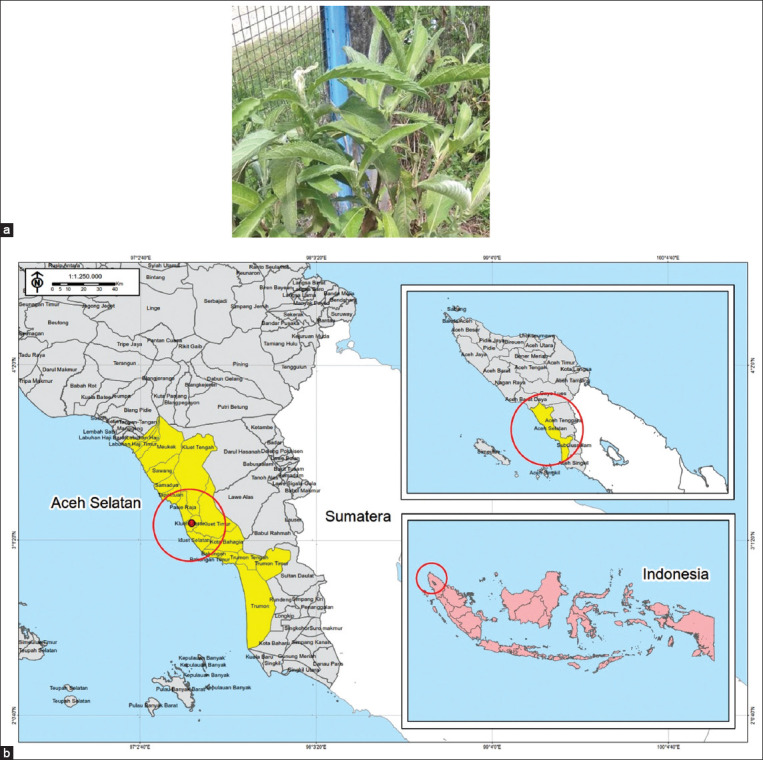
(a) Appearance of capa (*Blumea balsamifera*), (b) the sampling location [Source: Indonesian Earth Map (RBI), BIG 2020 and Administrative Map, BAPPEDA Aceh 2013-2033].

The study was carried out from September 2020 to January 2022. *Blumea balsamifera* leaves were taken from Gunongpulo village, South Aceh District, Aceh Province, Indonesia, at the coordinate of 03° 07'13.7” N and 97° 20' 28.2” E. The shape of the *B. balsamifera* is shown in Figure-1a and the map of the sampling location can be seen in Figure-1b.

### Plant identification

Plant identification was carried out at the Biology Laboratory Faculty of Mathematics and Natural Sciences of Syiah Kuala University, Banda Aceh, and the result showed that the plants belong to the genus Blumea with the species *B. balsamifera* L, as stated in the letter of identification result Number B/647/UN11.18.1/TA/00.01.2020.

### Characteristics sample of *B. balsamifera*

The plants grow wild in settlements and house yards. We identified the *B. balsamifera* plants based on the following characteristics: A height of >4 m; dark green upright round stems with a diameter of 3–5 cm; hairy and aromatic top. A single leaf has a height of 6–30 cm and a width of 1.5–12 cm with an oval shape, sharp edges, serrated loin, pinnate leaves, and 2–3 additional leaves on the petiole. The surface of the upper leaves is slightly rough, while the undersides of the leaves are tight and smooth.

### Preparation of the *B. balsamifera* leaf extract

The *B. balsamifera* leaf extract preparation consisted of several steps. First, we cleaned the leaves to remove any insects, dirt, and other plants attached to them. Then, we washed and dried them without exposing them to direct sunlight. Next, we ground the dried leaves using a blender to obtain a powder. We then placed 2000 g of the powder in a container, immersed it in 1000 mL of ethyl acetate, and let it macerate for 24 h. After that, we filtered the extracts using filter paper to obtain the filtrate. We then soaked the residues again with the solvent and repeated the process until the solvent became colorless. Finally, we dried the extracts using a vacuum rotary evaporator at 50°C to get viscous semi-solid masses.

### Formulation of the *B. balsamifera* leaf gel with Carbopol 940

The formulation of the *B. balsamifera* leaf gel using Carbopol 940 comprised several steps. First, we put 0.5 g of Carbopol 940 into a beaker and added 70 mL of distilled water. We then stirred the solution using a magnetic stirrer at 80°C for 30 min. Next, we ground the Carbopol base into a mortar and mixed it with 1 g of triethanolamine and 15 g of propylene glycol. We then stirred the substances evenly. Subsequently, we added 0.2 g of methylparaben and homogenized the gel base. We added various concentrations of *B. balsamifera* leaf extract, as shown in Table-1. We also added 30 mL of distilled water to obtain 100 mL gel. Finally, we stirred the gels until they became homogenous, stored them in a closed container and allowed them to stand for 24 h to cool down and assess their stability [25, 29]. We evaluated stability once a week for 1 month.

**Table-1:** Ingredients used for the gel formulation.

Formulation	K0 (g)	K1 (g)	K2 (g)	K3 (g)	K4
*Blumea balsamifera* extract	0	2,5	5	10	Gentamicin 1%
Carbopol 940	0.5	0.5	0.5	0.5	
TEA	1	1	1	1	
Propyleneglycol	15	15	15	15	
Methylparaben	0,2	0,2	0,2	0,2	
Aquadest add	Add to 100	Add to 100	Add to 100	Add to 100	

K0=A negative control group - Gel base. K1=A group using gel with 2.5% *B. balsamifera* leaf extract. K2=A group using gel with 5% *B. balsamifera* leaf extract. K3=A group using gel with 10% *B. balsamifera* leaf extract. K4=A positive control group – Gentamicin 1%. TEA=Triethanolamine

### Experimental animals

This study used 30 male white rats (*Rattus norvegicus*) aged 2–3 months and weighing 150–200 g from the UTD Laboratory of Experimental Animals, Faculty of Veterinary Medicine, Syiah Kuala University, Banda Aceh. We determined the necessary sample size using the Federer formula: (n−1) (t−1) ≥ 15, (n−1) (5−1) ≥ 15, n 19/4 = 4.75. The calculated sample size was 5.27. Thus, we used six rats in each group and a total of 30 rats for the study.

During the 1-week adaptation period, all rats were placed together in a cage and fed with a standard diet (T79-4 type) *ad libitum*. After the adaptation period, we separated the rats in treatment and control groups. The cage of each treatment group was equipped with drinking water and rations [30]. Before grouping, we measured the rats’ baseline weights [31].

### Incision

We made an incision on the rats’ back skin parallel to the os. vertebrae and 5 cm from the ear, on a previously sheared area. The incision had a diameter of 3 cm and a subcutaneous depth. To reduce the pain, we applied ketamine-xylazine as general anesthesia on the skin area to be incised. Then, we disinfected the skin with 70% alcohol. Finally, we separated the rats into five treatment groups.

### Gel application and wound healing evaluation

We treated the incisions of each of the five groups with a different gel: K0 (gel base without *B. balsamifera* leaf extract), K1 (gel with 2.5% *B. balsamifera* leaf extract), K2 (gel with 5% *B. balsamifera* leaf extract), K3 (gel with 10% *B. balsamifera* leaf extract), and K4 as a positive control (1% gentamicin). We then assessed wound healing by measuring wound length, histologically analyzing skin tissue, and quantifying IL-2. We observed the histological appearance of the rats’ treated skin through a microscope on days 7 and 14 of the treatment and searched for fibroblasts and angiogenesis in the wound tissues. Finally, we compared the groups with each other.

### Statistical analysis

Results are expressed as means ± standard deviation. Comparisons between the groups were performed using a one-way analysis of variance (ANOVA) followed by least significant difference post hoc test with SPSS 22.0 (IBM Corp., NY, USA). p < 0.05 was considered to be statistically significant.

## Results and Discussion

### Macroscopic evaluation of wound length reduction

**Figure-2:**
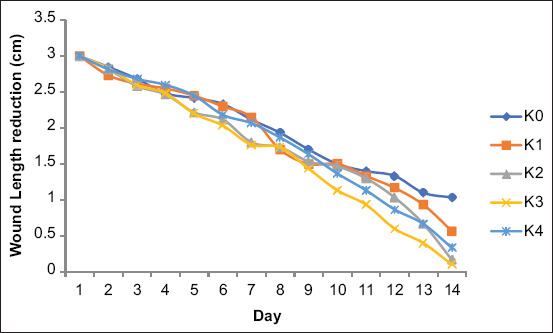
Comparison of mean wound length reduction.

The wound length evaluation showed that the K3-treated group had the greatest average reduction in wound length; Figure-2 displays the comparison chart of the treatment groups.

The one-way ANOVA on wound length reduction revealed a significant difference between treatment groups (p < 0.05). The K3-treated group (gel containing 10% *B. balsamifera* leaf extract) displayed the greatest incision length reduction. The length had decreased by 1.4 ± 0.081 cm on day 7 and 2.9 ± 0.081 cm on day 14 after treatment. Meanwhile, the incisions of the K2-treated group (gel with 5% *B. balsamifera* leaf extract) had decreased by 1.33 ± 0.124 cm on day 7 and 2.833 ± 0.124 cm on day 14. Interestingly, the K3- and K2-treated groups showed better results than the positive control group (treated with 1% gentamicin ointment), which had an average decrease in incision length of 1.067 ± 0.094 cm on day 7 and 2.667 ± 0.169 cm on day 14. Table-2 presents the results of the ANOVA on wound length incision reduction.

**Table-2:** Results of ANOVA test on wound length reduction.

Day of treatment	Wound length reduction	Sum of squares	df	Mean square	F	Sig.
7 day	Between groups	0.789	4	0.197	4.698	0.022
	Within groups	0.420	10	0.045		
	Total	1.209	14			
14 day	Between groups	1.709	4	0.427	4.931	0.019
	Within groups	1.867	10	0.087		
	Total	2.576	14			

ANOVA=Analysis of variance

Our results are in line with the literature. A previous study conducted by Huang *et al*. [21] showed that a 92 mg/kg body weight *B. balsamifera* leaf extract using water as a solvent reduced edema by up to 6.4%. This study reveled that *B. balsamifera* leaf ethanol extract with concentrations of 40%, 60%, 80%, and 100% inhibited the growth of *Escherichia coli* and *Staphylococcus aureus* with inhibition zones of 14–23 mm and 17–21 mm, respectively. Besides, *B. balsamifera* leaf extract potentially has antimicrobial activity [32]. Finally, a 2.5 g/kg body weight dose of *B. balsamifera* leaf extract had a positive effect on wound healing in white rats; indeed, it was nearly as effective as the positive control, followed by the 0.6 g/kg and 1.25 g/kg body weight dose of *B. balsamifera* leaf extracts [33]. Thus, our results showed that the *B. balsamifera* extract gel enhanced wound healing.

### Histological evaluation of wound healing

**Figure-3:**
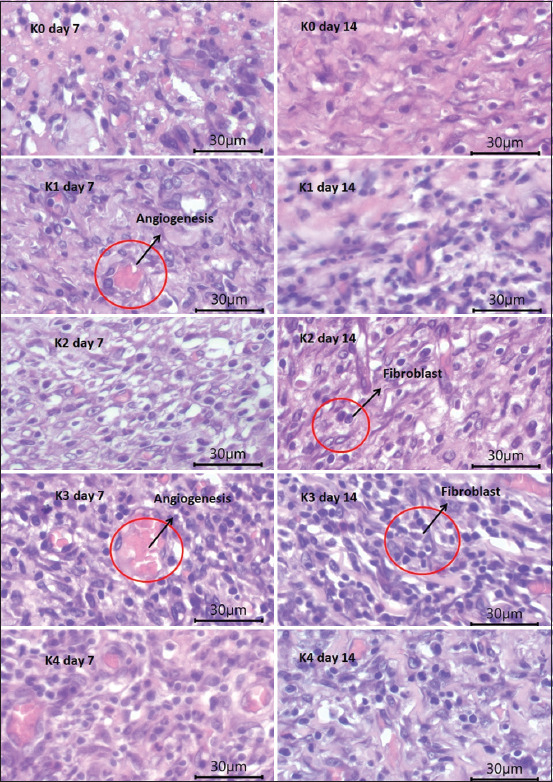
Histology of fibroblasts and skin angiogenesis on 7 and 14 days.

We counted fibroblasts and angiogenesis marks in five visual fields with 40 times magnification. Figure-3 shows a comparison in the number of fibroblasts and angiogenesis marks 7 and 14 days after treatment. Figure-3 shows fibroblast growth and angiogenesis progression in skin tissues of rats treated with the gels. In the red circles, the blue spots indicate the newly formed fibroblast network and the reddish spots indicate angiogenesis or blood vessel network formation. To heal wounds, skin cells need both fibroblasts and angiogenesis. The more fibroblasts and angiogenesis, the faster the wound heals.

We compared fibroblast cell density and angiogenesis mark counts in the incision skin tissues of white rats in the five different treatment groups. The K3-treated group had clearly more fibroblasts and angiogenesis marks than the other groups. We observed the tissues with a microscope to determine the most effective treatment. Table-3 displays the fibroblast and angiogenesis mark counts in five visual fields with 40 times magnification.

**Table-3:** Average number of fibroblasts and angiogenesis.

Treatment group	Fibroblast		Angiogenesis	
	
	Mean day 7 ± SD	Mean day 14 ± SD	Mean day 7 ± SD	Mean day 14 ± SD
K0	15.800 ± 0.748	19.200 ± 2.355	2.267 ± 0.377	2.667 ± 0.659
K1	18.200 ± 0.748	15.867 ± 0.942	3.000 ± 0.588	2.800 ± 0.282
K2	20.067 ± 0.736	25.667 ± 2.379	2.667 ± 0.249	2.933 ± 0.188
K3	25.933 ± 1.388*	27.933 ± 2.288*	3.933 ± 0.377*	4.000 ± 0.282*
K4	20.800 ± 1.232	27.333 ± 2.763	3.400 ± 0.489	3.400 ± 0.326

* The highest number of fibroblast and angiogenesis was in the K3 treatment group after 7 days and 14 days. SD=Standard deviation

**Figure-4:**
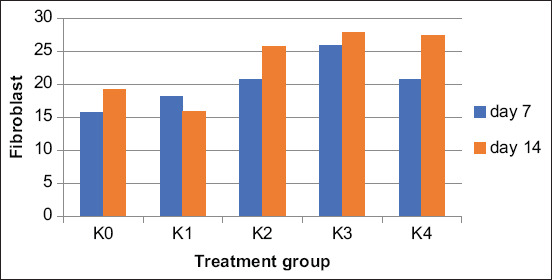
Comparison of number fibroblast cells between treatment groups after 7 and 14 days treatment.

**Figure-5:**
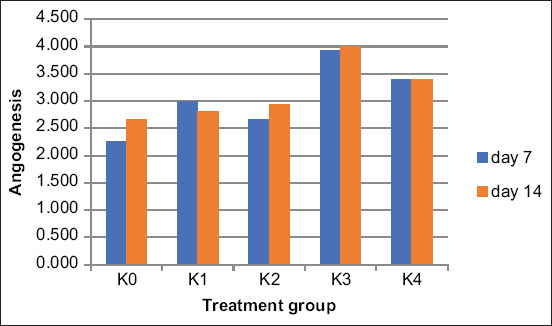
Comparison of number angiogenesis between treatments group after 7 and 14 days treatment.

The K3-treated group (10% *B. balsamifera* leaf extract gel) had the highest average number of fibroblasts (25.933 ± 1.388), surpassing that of the positive control group (20.800 ± 1.232) on day 7. On day 14, the K3-treated group also had the highest average number of fibroblasts (27.933 ± 2.288). Meanwhile, the K2-treated group (5% *B. balsamifera* leaf extract) and the positive control group had similar counts (25,667 ± 2.379 and 27.333 ± 2.763, respectively). Figures-4 and 5 show each group’s average number of fibroblasts and angiogenesis marks after 7 and 14 days of treatment.

Angiogenesis is the growth of blood vessels in skin tissues. The K3-treated group also displayed the highest angiogenesis mark count (3.9 on day 7 and 4.00 on day 14). The ANOVA showed that the data were homogeneous, and the differences between groups were significant (p < 0.05). Table-4 details the ANOVA results.

**Table-4:** Results of ANOVA test on fibroblast and angiogenesis.

Day of treatment	Sum of squares	df	Mean square	F	Sig.
Fibroblast					
7 days					
Between groups	169.803	4	42.451	27.685	0.000
Within groups	15.333	10	1.533		
Total	185.136	14			
14 days					
Between groups	346.053	4	86.513	11.549	0.001
Within groups	74.907	10	7.491		
Total	420.960	14			
Angiogenesis					
7 days					
Between groups	4.997	4	1.249	4.462	0.025
Within groups	2.800	10	0.280		
Total	7.797	14			
14 days					
Between groups	3.563	4	0.891	4.024	0.034
Within groups	2.213	10	0.221		
Total	5.776	14			

ANOVA=Analysis of variance

Thus, our results confirmed our research hypothesis: The active compounds of *B. balsamifera* leaves enhance wound healing, especially in a topical gel form.

The fibroblast growth factor is an angiogenic factor that can also form a complex with heparin, increasing the latter’s cell activation efficiency after the fibroblast growth factor binds to its receptor [34]. The increase in fibroblasts can accelerate wound healing. Angiogenic factors can be categorized into three groups: The first group consists of angiogenic factors that target endothelial cells to stimulate mitosis [35]. The second group is molecules that activate a broad range of target cells other than endothelial cells. Several cytokines, chemokines, and angiogenic enzymes belong to this group. Fibroblast growth factor 2 was the first cytokine of this group to be characterized. The third group is indirect factors. The angiogenic factors in this group are produced by macrophages and endothelial cells [36]. Angiogenesis plays a role in physiological and pathological processes. Angiogenesis is a natural physiological process tightly regulated; in a healthy body, it is crucial to wound healing.

### Evaluation of IL-2 levels in rat blood serum

**Figure-6:**
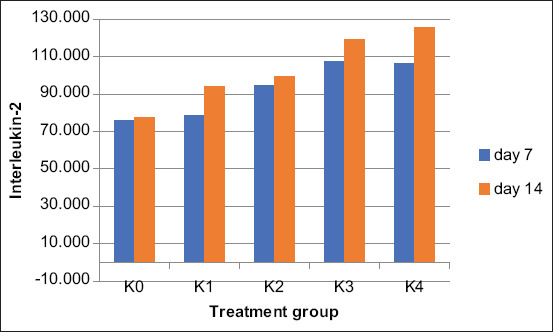
Comparison of levels interleukin-2 between treatments group.

We quantified the IL-2 levels in the blood serum of all the experimental animals by enzyme-linked immunosorbent assay. Figure-6 compares the IL-2 levels of each group.

Table-5 presents the average IL-2 levels in the blood serum of white rats treated with gel for 7 and 14 days.

**Table-5:** Average IL-2 cytokine levels in the blood serum of rats.

Treatment group	Average of IL-2 (in ng/l)	

	Mean day 7 ± SD	Mean day 14 ± SD
K0	76.100 ± 3.290	77.390 ± 3.391
K1	78.576 ± 8.380	94.106 ± 11.832
K2	94.420 ± 8.322	99.603 ± 13.889
K3	107.776 ± 6.673*	119.190 ± 12.260
K4	106.563 ± 5.657	125.740 ± 12.081*

* The highest levels of IL-2 was in the K3 treatment group after 7 days and K4 after 14 days. IL-2=Interleukin-2, SD=Standard deviation

The K3-treated and positive control groups had the highest blood serum IL-2 levels on day 7 and day 14, respectively. Therefore, the gel with 10% *B. balsamifera* leaf extract used in K3 was effective for wound healing. This experiment confirmed the results obtained by measuring the reduction in incision length and quantifying fibroblasts and angiogenesis in the wound tissues. IL-2 is a growth factor accelerating cell tissue growth, and thus wound healing. The higher the IL-2 levels, the faster the wound healing process. The ANOVA yielded significance values of 0.002 on day 7 and 0.010 on day 14 (p < 0.05). Thus, the treatment groups had significantly different IL-2 levels. Table-6 summarizes the ANOVA of IL-2 levels.

**Table-6:** The average wound length reduction.

Treatment Group	Reduction in incision length (in cm)	

	Mean day 7 ± SD	Mean day 14 ± SD
K0	0.800 ± 0.216	1.967 ± 0.410
K1	0.933 ± 0.249	2.433 ± 0.262
K2	1.333 ± 0.124	2.833 ± 0.124
K3	1.400 ± 0.081*	2.900 ± 0.081*
K4	1.067 ± 0.094	2.667 ± 0.169

* The highest reduction in wound length was in the K3 treatment group after 7 days and 14 days

*B. balsamifera* leaves can also reduce polyethylene glycol-2 and protein translation. They also effectively reduce the levels of inflammatory cytokines, such as IL-1, IL-6, and tumor necrosis factor a [19, 20]. Soluble protein cytokines produced by lymphocyte cells, such as IL-2, function as intracellular signals and regulate nearly all cellular processes, such as growth, proliferation, and differentiation. IL-2 is known as a cell growth factor regulating the balance of activated lymphocytes. Under normal conditions, T helper 1 cells produce IL-2 after their activation by antigens or mitogens. However, its maximum production requires secondary messengers [37]. The significance was 0.002 in the 7-day treatment group and 0.010 in the 14-day treatment group (Table-7). These values indicated that there were significant differences in IL-2 cytokine levels in each treatment group. In line with the previous study of Fan *et al*. [19], this study proved that gels containing various *B. balsamifera* leaf extract concentrations significantly increased the IL-2 levels in the blood serum of white rats.

**Table-7:** Results of ANOVA test on cytokine – IL-2 levels.

Day of treatment	IL-2 levels	Sum of squares	df	Mean square	F	Sig.
7 days	Between groups	2629.452	4	673.113	9.890	0.002
	Within groups	680.569	10	68.057		
	Total	3373.021	14			
14 days	Between groups	4576.537	4	1144.134	5.952	0.010
	Within groups	1922.118	10	192.212		
	Total	6498.655	14			

IL-2=Interleukin-2, ANOVA=Analysis of variance

## Conclusion

Gels with 2.5%, 5%, and 10% *B. balsamifera* leaf extract enhanced incision wound healing in white rats. The 10% *B. balsamifera* leaf extract gel (K3) was the most effective, followed by 5% and 2.5% *B. balsamifera* leaf extract gels (K2 and K1, respectively). We assessed the effectiveness of these gels was measured based on three wound healing parameters: The decrease in the incision wound length, histological evaluation of fibroblasts and angiogenesis, and IL-2 quantification in the blood serum of the animals treated for 7 and 14 days. Various active compounds of the *B. balsamifera* leaves, such as borneol, jasmoline, and caryopelline, can heal wounds more effectively when formulated into a gel.

## Authors’ Contributions

MM: Contributed to conceptualization, methodology, investigation, formal analysis, Writing-original draft. MH: Contributed to conceptualization, methodology, validation, funding acquisition, supervision. MarM: Contributed to data curation, writing review and editing. SU: Contributed to project administration, supervision, visualization; validation, writing review and editing. All authors have read and approved the final manuscript.
